# De Novo Transcriptome Profiling of Mustard Aphid (*Lipaphis erysimi*) and Differential Expression of Transcripts Associated with Feeding and Non-Feeding Conditions and Developmental Stages

**DOI:** 10.3390/insects15090682

**Published:** 2024-09-09

**Authors:** Rubina Chongtham, Manvi Sharma, Rohit Nandan Shukla, Gopal Joshi, Amar Kumar, Shailendra Goel, Manu Agarwal, Arun Jagannath

**Affiliations:** 1 Department of Botany, University of Delhi, Delhi 110007, India; 2 Department of Botany, Deshbandhu College, University of Delhi, Delhi 110019, India; 3 Bionivid Technology Pvt. Ltd., Kasturi Nagar, Bengaluru 560043, India

**Keywords:** *Lipaphis erysimi*, transcriptome, feeding, non-feeding, adult, nymph, effectors

## Abstract

**Simple Summary:**

Aphids are phloem feeders and are one of the most destructive pests of crop plants. *Lipaphis erysimi* is a specialist aphid that infests members of the Brassicaceae family and has detrimental impacts on the yield of oilseed and vegetable Brassicas in the Indian subcontinent. Recognising the critical need for developing aphid control strategies, we aimed to enhance the pool of gene targets for RNAi for developing resistance in Brassica crop plants against *L. erysimi* infestation. To understand the variations in expression profiles of genes while the aphid is feeding upon the host plant, as well as between two developmental stages (adults and nymphs), we conducted a two-way transcriptome-based differential expression analysis between feeding and non-feeding adults and between adults and nymphs. This led to the identification of important transcripts involved in detoxification, feeding, metabolism and aphid development. Our study provides valuable insights into the molecular mechanisms underlying feeding and development in aphids. It also generated an important resource for further research on aphid biology and for developing effective RNAi-based strategies for pest management and control.

**Abstract:**

*Lipaphis erysimi* is a specialist aphid of the Indian subcontinent that causes significant yield losses in oilseed Brassicas. Several aphid genes have been used as preferred targets in RNAi-based transgenic plants for aphid resistance. In order to enhance the repertoire of potential target genes for aphid control and to identify the genes associated with aphid feeding and development, we performed a two-way comparative study of differential gene expression profiles between (i) feeding and non-feeding adults and (ii) adult and nymph developmental stages of *L. erysimi.* De novo RNA-seq of aphids using Illumina technology generated a final transcriptome comprising 52,652 transcripts. Potential transcripts for host selection, detoxification, salivary proteins and effectors, molecular chaperones and developmental genes were identified. Differential gene expression studies identified variations in the expression of 1502 transcripts between feeding and non-feeding adults and 906 transcripts between nymphs and adults. These data were used to identify novel target genes for RNAi-based aphid control and facilitate further studies on the molecular basis of aphid feeding and development.

## 1. Introduction

Aphids are phloem feeders of the Order Hemiptera and are one of the most devastating pests of cultivated crops globally. There are over 5000 species of aphids [1], of which ~450 species are pests of crop plants [2]. Aphids also function as vectors for transmission of almost 30% of plant viruses [3]. In addition to causing yield losses, viral manipulation of phytohormone signalling in infested plants also influences vector–plant interactions [4,5,6]. *Lipaphis erysimi,* also known as mustard aphid, is a Brassicaceae specialist and is one of the most significant challenges to the cultivation of the oilseed crop *Brassica juncea* (Indian mustard) in the Indian subcontinent [7]. *B. juncea* accounts for ~90% of the acreage under rapeseed–mustard cultivation in India [8]. *L. erysimi* not only causes extensive losses in yield (65–96%) and a reduction in oil content (up to 15%) but also reduces crop productivity indirectly by transmitting disease-causing plant viruses [9,10,11].

Aphid–plant interactions are complex and initiate with the identification and recognition of a host plant based on visual, olfactory and gustatory cues, followed by host acceptance, sustained phloem feeding and rapid parthenogenetic multiplication of the insect [12,13,14,15]. The adaptations of aphids to a phloem diet, their ability to overcome plant defences and their rapid rate of reproduction contribute to their successful infestation of plants. Plants employ different modes of defence against aphid infestation, including physical barriers, viz., waxy cuticle [16], glandular trichomes [17], alteration and remodelling of the cell wall [18], production of defence molecules, including secondary metabolites such as glucosinolates [19] and enzymes such as proteases [20], and protease inhibitors [21]. Injury to phloem by aphids is mitigated by phloem proteins [22] and callose plugs [23]. Additionally, plants may release volatiles to attract natural predators of aphids [24] and produce reactive oxygen species (ROS), leading to a hypersensitive response (HR). However, aphids have evolved well-adapted mechanisms to counter these defences. They have specialised piercing-sucking mouthparts with long and slender stylets that achieve intercellular penetration and feeding from phloem with minimal physical injury to the plant [25]. Although these sap-sucking pests induce local, as well as systemic, plant resistance by releasing elicitors, aphids can also suppress local plant defences by releasing salivary proteins that bind Ca^2+^ ions, which are required for phloem occlusion and an oligogalacturonide-induced plant defence response [26,27]. Aphid salivary proteins are involved in the degradation of plant proteins, detoxification of plant defence molecules and interference with plant signalling pathways [28].

Expression analyses of the genes involved in the above processes can provide important cues to control aphid infestation. Aphids of different developmental stages have been demonstrated to show different transcriptomic responses to host plants in terms of detoxification, apart from the expected differences in cuticle formation and hormone-related transcripts [29]. Transcriptome studies have been used to understand the molecular basis of development, aphid–plant interactions and aphid–endosymbiont interactions on the model pea aphid, *Acyrthosiphon pisum*. *A. pisum* has been used to study the differential expression of wing development genes [30] and identification of secretory and effector proteins [28]. Transcriptome studies have also been performed in some non-model aphids, viz., the soybean aphid (*Aphis glycines*) [31] and grain aphid (*Sitobion avenae*) [32]. Changes in the expression levels of proteins, viz., heat shock proteins (HSPs) and Cyt P450s, were shown to aid in the adaptation of cotton aphid to different stages of growth and development of its host [33].However, transcriptome studies on plant–aphid interactions have primarily focussed on changes in the host plant transcriptome during compatible and non-compatible interactions with aphids [34,35,36]. A comprehensive analysis of transcriptomic changes within the aphid during such interactions with its host is lacking. Moreover, the differential expression of aphid genes during feeding and non-feeding conditions has not been studied until now. There is also limited information on gene expression changes during development from nymphs to adults. Ji et al. (2016) [29] conducted a study on the generalist aphid *M. persicae* and identified 2244 differentially expressed genes between adults and nymphs that were related to cuticle formation, detoxification and metabolism.

In the present study, we performed de novo transcriptome sequencing of a specialist aphid, *L. erysimi*, to investigate differential expression profiles between feeding adults (Adult Feeding—AF) and non-feeding adults (without access to food for three hours; Adult Non-Feeding—ANF) and between different developmental stages (adults and nymphs). To the best of our knowledge, this is the first report on global transcriptomic differences between normal feeding and non-feeding conditions in any aphid and outlines the possible roles of different sets of genes during feeding conditions and during the short-term non-availability of food.

## 2. Materials and Methods

### 2.1. Maintenance of Aphids

*L. erysimi* cultures consisting of parthenogenetic apterous females were initiated from a single cohort of apterous female adults (provided by Prof. A. K. Singh, Department of Zoology, University of Delhi) and propagated in a culture room at 21 ± 1 °C with a photoperiodic cycle of 16 h light:8 h dark and a relative humidity (R.H.) of ~55–70%. The cultures were maintained on excised *B. oleracea* (cauliflower) leaves placed over moistened blotting paper in transparent plastic boxes. The leaves were changed every alternate day, distributing progenies to new boxes to prevent nutritional stress and crowding in order to maintain a uniformly apterous culture.

### 2.2. Collection of Biological Material

Three types of biological samples were collected for transcriptome sequencing: (i) Adult Feeding (AF)—comprising adult apterous aphids that were allowed to feed normally, (ii) Adults starved for 3 h (Adult Non-Feeding) (ANF) and (iii) Nymph Feeding (NF)—comprising feeding nymphs of all four nymphal stages (N1–N4) pooled together. Each sample consisted of ~300–400 apterous morphs. Segregation of the adult and nymphal stages was done under a Stemi DV4 microscope (Zeiss, Oberkochen, Germany). The AF and NF samples were harvested directly from *B. oleracea* leaves, and only those aphids were harvested that had their stylets inserted and were feeding. For the ANF sample, apterous adult aphids were segregated in empty plastic boxes, subjecting them to non-feeding conditions for 3 h. All samples were flash frozen in liquid nitrogen and stored at −80 °C until further use.

### 2.3. Preparation of cDNA Libraries, Sequencing and Data Analysis

**Figure 1:**
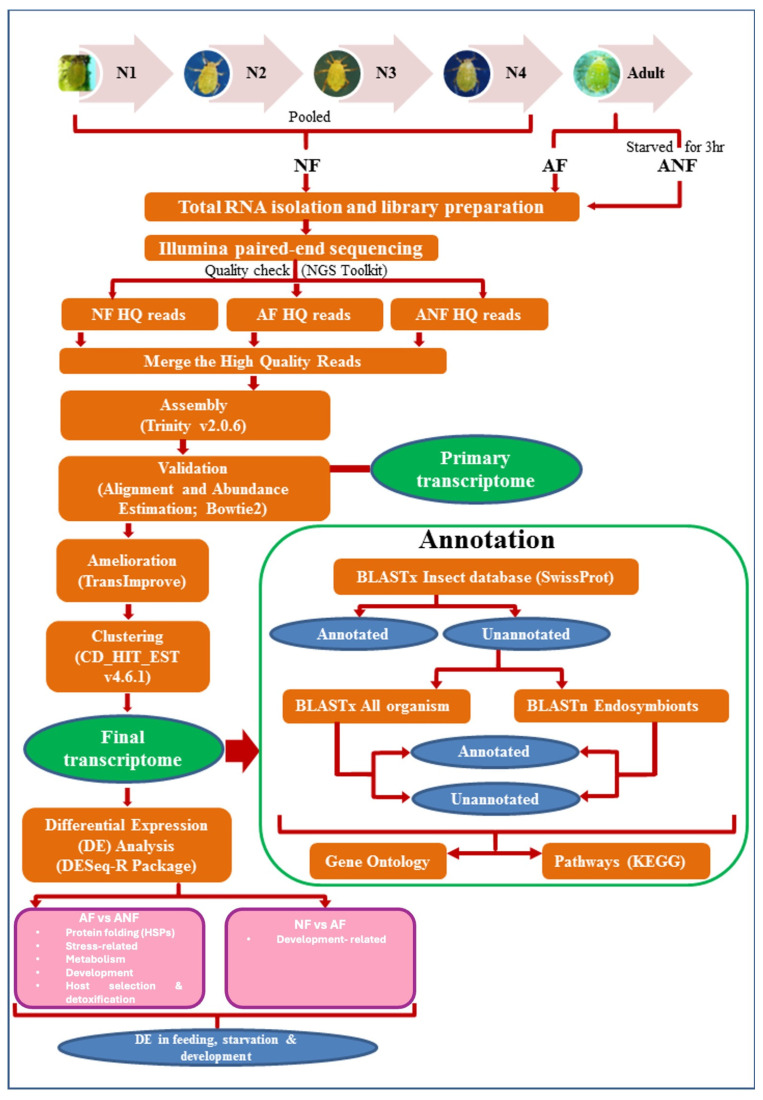
Flowchart representing the workflow for the transcriptome analysis of *L. erysimi*. The tools used at different steps are given within the parentheses. N1–4: nymphal stages 1 to 4, NF: Nymph Feeding, AF: Adult Feeding, ANF: Adult Non-Feeding—starved for 3 h and HQ: high-quality.

Total RNA was isolated from each sample using the Trizol method [37]. cDNA libraries were prepared from 2 µg total RNA per library using the TruSeq RNA Sample Prep Kit v2 (Illumina Inc., San Diego, CA, USA), according to the manufacturer’s instructions. The complete pipeline used for the analysis is given in Figure 1. The quality of the cDNA libraries was assessed using 2100 Bioanalyzer (Agilent Technologies, Santa Clara, CA, USA). The libraries were sequenced with the Genome Analyzer IIx and HiSeq 2000 (Illumina, USA) sequencer using the paired-end method, with an expected average read length of ~100 bp. The raw reads were subjected to quality checks using the NGS QC toolkit [38], in which the adapter sequences were trimmed and low quality reads (with read lengths < 70% and a Phred score < 20) were removed. The resulting high-quality filtered paired sequence reads from the adult feeding, adult non-feeding and nymph feeding samples were provided as an input for transcriptome assembly. De novo transcriptome assembly and validation was performed using Trinity assembler [39]. The primary transcriptome assembly thus obtained was validated by mapping the HQ filtered reads back to the assembled reads using the Bowtie2 tool [40]. Transcript abundance was estimated for each sample using the RSEM tool [41]. The transcriptome data were further ameliorated using TransImprove (Bionivid Technology Pvt. Ltd., Bengaluru, Karnataka, India) to generate the final transcriptome assembly. The final transcriptome was clustered with CD-HIT-EST (v4.6.1) [42]. To assess the completeness of the assembly, BUSCO (Benchmarking Universal Single-copy Orthologs) (v5.7.1) [43] analysis was conducted against the insecta_odb10 database. Normalisation, differential gene expression analysis and biological significance analysis were completed using the DESeq-R package [44]. Annotation for the final transcripts longer than 200 bp was completed using a BLASTx identity search [45] against all insect protein sequences from Swiss-Prot. The remaining unannotated transcripts were aligned against the protein sequences of all organisms in the NCBI non-redundant database using BLASTx. BLASTn was performed for the unannotated transcripts against sequences of bacterial endosymbionts (listed in *Buchnera*Base) in the NCBI nucleotide database. Blast hits were filtered for transcripts with E-value scores ≤ 10 × 10^−4^. Gene Ontology (GO) annotation and pathway analysis of the transcripts was performed based on the Kyoto Encyclopedia of Genes and Genomes (KEGG) database. Transcripts with P_adjusted_ values < 0.05 were considered differentially expressed. The transcriptome was analysed to identify transcripts with signal peptides (SignalP4.1), transmembrane domains (TMpred, http://www.ch.embnet.org/software/TMPRED_form.html (accessed on 1 August 2024)) and nuclear localisation signals (SeqNLS, http://mleg.cse.sc.edu/seqNLS (accessed on 1 August 2024)) [46].

### 2.4. Real-Time Quantitative PCR

For validation of the digital expression data (refers to the expression data that we obtained from transcriptome sequencing, i.e., after quantifying, normalising reads and performing DEGs analysis), the total RNA was isolated from each of the three samples described above in three independent biological replicates, each of which had two technical replicates. Five micrograms of each RNA sample was treated with 2U DNAseI (New England Biolabs) at 37 °C for 30 min, followed by phenol-chloroform-isoamyl alcohol extraction, sodium acetate-ethanol precipitation and wash with 70% ethanol. The RNA integrity was checked on a 1.2% denaturing agarose gel. cDNA was prepared from 500 ng of each DNA-free RNA sample using an iScript Reverse transcription kit (Bio-Rad Laboratories, Inc., Hercules, CA, USA) following the manufacturer’s protocol. The pea aphid *Elongation Factor 1α* (*EF1α*) was found to show stable expression levels in all the three samples of the current study and was used for normalisation. The *EF1α* primers used were 5′ ATGGAATGGAGACAACATGTTGG 3′ (forward primer) and 5′ AGAGCCTTGTCAGTTGGGCG 3′ (reverse primer). PCRs were done on a CFX Connect Real-Time System (Bio-Rad) in 96-well plates using SYBR Green (Roche) for the selected transcript sequences. Primer sequences for the validated transcripts are provided in Table S1. After normalisation using *EF1α* (∆Ct), relative fold changes were calculated using the ∆∆Ct method by pairwise comparison of adult feeding (AF) (control) with nymph feeding (NF)/adult non-feeding (ANF) (Test), depending on the transcript.

## 3. Results and Discussion

### 3.1. Analyses of Sequencing Data and De Novo Transcriptome Assembly

The three sets of samples of apterous aphids, viz., AF, ANF and NF, were collected and used for the construction of transcriptome sequencing libraries. While the AF and NF samples were collected directly from *B. oleracea* leaves, the ANF samples were starved for 3 h prior to harvesting. It has been shown in earlier studies that the threshold duration of phloem feeding indicating acceptance of the host is ≥10 min [47]. An earlier study on *M. persicae*, which is a generalist aphid that also infects Brassicas, showed that the effects of nutrition loss on aphid growth due to starvation for <4 h were overcome once returned to the host plants but not when the starvation period exceeded four hours [48]. Thus, an induced non-feeding state by starvation for 3 h was followed in this study to ensure the arrest of aphid activities involved in aphid–host interactions without causing irreversible damage to aphid health and survival.

As given in Figure 1, the transcriptome libraries were sequenced on a high-throughput Illumina sequencing platform. The number (in millions) of high-quality (HQ) paired-end filtered reads obtained were 165.69, 155.29 and 215.41 in the AF, ANF and NF samples, respectively (Table 1).

Due to the lack of genome sequence information of *L. erysimi*, de novo assembly of the transcriptome data was performed using Trinity assembler [39]. The HQ reads from all three sets of samples (536.40 million) were merged to generate the transcriptome assembly. A comparison of the primary transcriptome and the final ameliorated transcriptome is given in Table 2. The amelioration step improved the average transcript length and N50 value of the final transcriptome to 1064 and 1806, respectively. The average G + C content of the final *L. erysimi* transcriptome was low at 34.52%, which is in consonance with earlier studies on insect genomes [49,50]. A high BUSCO score of 96.8% indicated completeness and good quality of the assembly (Table 3).

### 3.2. Annotation of Assembled Transcripts of L. erysimi and Its Endosymbiont, Buchnera aphidicola

**Figure 2:**
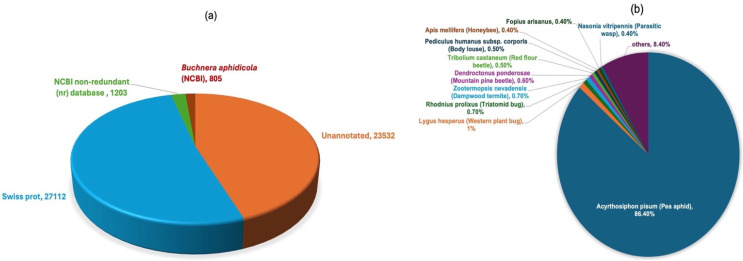
(**a**) Distribution of transcripts according to their BLAST results against the respective databases. (**b**) Distribution of transcripts among the top 10 insect species, according to their BLAST results against the insect database.

The 52,652 transcripts obtained after the final assembly were subjected to BLASTx-based annotation against insect protein sequences in the Swiss-Prot database. A total of 27,112 transcripts (50.5%) were annotated with insect proteins from Swiss-Prot (Table S2), of which 96.13% had E-values ≤ 10^−5^ (Figure 2a). More than 86% of these insect-annotated transcripts (23,429) comprised *A. pisum* sequences. The top ten species distribution of the annotated transcripts is given in Figure 2b and Table S2. *Buchnera aphidicola* is a common bacterial endosymbiont that resides within aphids in specialised cellular structures called bacteriocytes [51]. These endosymbionts provide aphids with essential amino acids that are lacking in phloem sap [52]. Therefore, the remaining 25,540 unannotated transcripts were subjected to two independent BLAST analyses: BLASTn against the sequenced endosymbionts listed in *Buchnera*Base and BLASTx against the NCBI non-redundant (nr) database. This resulted in the annotation of 816, 128 and 1064 transcripts with endosymbionts (including 336 sequences from *B. aphidicola*), *A. pisum* and sequences of other organisms, respectively. Nikoh and Nagabachi (2009) [53] also reported the presence of *B. aphidicola* genes in the aphid *A. pisum.* Their results indicated that such genes were acquired from the bacterium by lateral gene transfer and are essential for the maintenance of the bacterial endosymbiont.

To ascertain the functional roles of transcripts, Gene Ontology (GO) and KEGG pathway distribution were analysed. GO analysis resulted in the classification of 17,296 transcripts by at least one of the three ontologies, i.e., biological process, molecular function and cellular component. Similarly, different pathways associated with transcripts were studied based on KEGG pathway categorisation, and the top five most abundant distribution of the KEGG pathways is given in Table 4. Pathway annotation identified 4128 insect-annotated transcripts, which were assigned to 165 different KEGG pathways. A significantly higher number of transcripts (1123, 27.2%) belonged to the category of metabolic pathways than others such as ribosome (257, 6.2%), RNA transport (190, 4.6%), spliceosome (138, 3.3%) and protein processing in the endoplasmic reticulum (136, 3.3%). A total of 23,532 transcripts did not show identity with any sequence available in the public domain and may therefore represent novel aphid transcripts/sequences that are unique to *L. erysimi*.

### 3.3. Differential Expression of Genes

Pairwise differential expression analysis of whole transcriptomes of all the samples identified genes that are differentially expressed under different conditions, i.e., feeding vs. non-feeding (AF vs. ANF) and in different developmental stages (NF vs. AF). Likewise, to look for significant differences (if any) among feeding nymphs and non-feeding adults, a similar analysis was conducted (NF vs. ANF). For the identification of differentially expressed transcripts in the studied samples, transcripts with log2 fold changes ≥ 2 or ≤−2 were considered significantly upregulated or downregulated, respectively, between the samples. These results are described and discussed in detail in the following subsections.

#### 3.3.1. Differentially Expressed Transcripts under Feeding and Non-Feeding/Starvation Conditions

**Figure 3:**
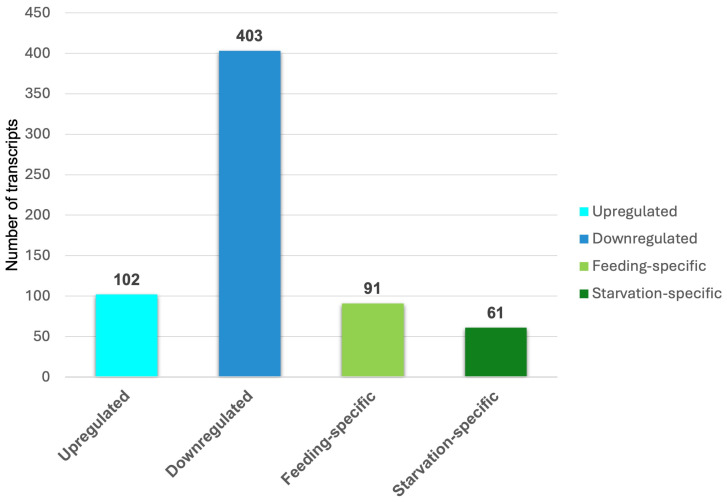
Number of differentially regulated, feeding- and starvation-specific/adult non-feeding transcripts between feeding adults and non-feeding adults. Upregulated and downregulated transcripts represent the higher and lower expression levels in feeding adults with respect to non-feeding adults.

In the comparison between the AF and ANF samples, 102 transcripts showed higher levels of expression in feeding adults (Figure 3; Table S3), and 403 transcripts showed higher expression in non-feeding adults (downregulated in AF vs. ANF; Figure 3; Table S4). There were 91 and 61 transcripts that were ‘specific’ to feeding and non-feeding aphids, respectively (Tables S5 and S6, respectively). In the case of the feeding-specific transcripts, 22 transcripts were ‘uncharacterised’, and 10 transcripts remained ‘unannotated’ that were concluded as ‘novel’ transcripts identified in our study. Similarly, in the starvation-specific transcripts, 15 transcripts were uncharacterised, and 20 transcripts were unannotated. Biologically, the ‘specific’ transcripts may include genes that are highly upregulated (or downregulated) in the relevant categories.

**Figure 4:**
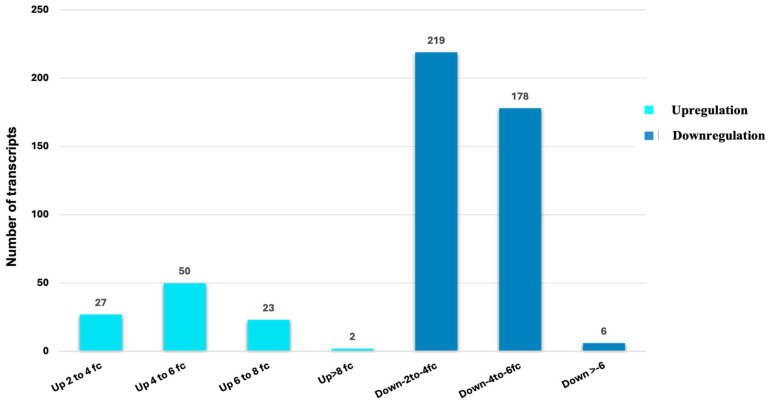
Distribution of differentially regulated transcripts in different categories of log2 fold changes. (up—upregulated and down—downregulated in feeding adults with respect to non-feeding adults, and fc—log2 fold changes).

The extent of up- or downregulation in differentially expressed transcripts and the distribution of transcripts in different ranges of up- or downregulation are shown in Figure 4. The maximum number of transcripts upregulated in adult feeding were in the range of 4 to 6 log2 fold changes, whereas those upregulated in non-feeding adults were in the range of −2 to −4 log2 fold changes. However, the number of downregulated transcripts (upregulated in non-feeding adults) was higher than the upregulated ones in feeding adults with respect to non-feeding adults. This observation may indicate a ‘normal’ condition of aphids that are feeding on host plants until they stop feeding, which leads to a drastic change in the expression profiles of the genes, as observed in the significantly higher number of upregulated transcripts in non-feeding adults.

#### 3.3.2. Biological Processes Associated with Differentially Expressed Genes in Feeding and Non-Feeding Conditions

**Figure 5:**
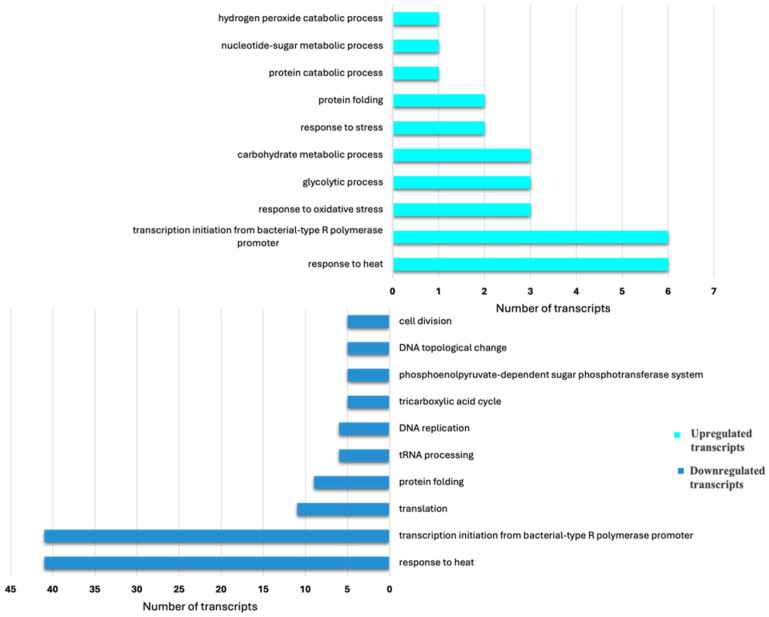
Classification of differentially expressed transcripts on the basis of Gene Ontology: Biological Process. The top 10 terms are represented for the comparison between AF (Adult Feeding) and ANF (Adult Non-Feeding). Upregulated and downregulated transcripts represent the higher and lower expression levels in feeding adults with respect to non-feeding adults.

The major biological processes influenced by feeding status are given by the ten most abundant GO terms for biological processes among the upregulated transcripts in feeding and non-feeding aphids (Figure 5).

‘Protein folding’ was one of the ten most abundant biological processes identified among the upregulated transcripts in both feeding and non-feeding aphids (downregulated in feeding adults) (Figure 5). All transcripts involved in protein folding were either HSPs or other chaperones. Out of the two upregulated transcripts involved in protein folding, one of the transcripts was HSP 83, which plays pleiotropic roles in the longevity, fecundity and embryogenesis of *A. pisum* [54]. On the basis of the BLASTx analysis, a total of 35 Hsps were identified in our study, including 2 that were specific to feeding adults, in addition to the upregulated ones. The roles of bacterial HSPs in the interactions of aphids with their biotic/abiotic environment have been described in earlier studies [55], and our study reveals the possibility of aphid chaperones playing an important role during feeding. Transcripts of the molecular chaperones with their expression patterns are given in Table S7.

It is observed that a greater number of transcripts are involved in ‘stress’ in feeding adults as compared to non-feeding adults (Table S7). During feeding, aphids encounter stress due to the activation of host defence mechanisms against herbivory, whereas, under non-feeding conditions, the stress is of a different nature. Another top GO term among the upregulated transcripts of feeding aphids was ‘response to oxidative stress’ (Figure 5). Earlier studies have shown the enhancement of detoxification enzymes in aphids in response to artificially elevated stressors in plants [56,57]. In our study, we found that such enzymes are overexpressed during routine feeding on host plants as well. Two such transcripts encoding catalase and cytochrome c peroxidase were identified. Catalase and cytochrome c peroxidase are involved in managing reactive oxygen species (ROS) as components of the antioxidant defence mechanism [58,59]. However, no such category of transcripts was reported in the non-feeding aphids. Therefore, the enhanced expression of transcripts involved in ‘response to oxidative stress’ might play an important role in preventing a hypersensitive response of the host plant to counter aphid feeding or oxidative stress from the ingested plant material. Other classes of proteins associated with response to oxidative stress identified in our study are glutathione-S-transferases (GSTs) and superoxide dismutases (SODs). Of the three SOD transcripts identified in this study, none showed significantly differential expression between feeding and non-feeding aphids. One GST transcript showed significant upregulation in non-feeding adults (downregulated in feeding adults). Therefore, some ‘detoxification’ enzymes identified in our data appear to be associated with the feeding state.

‘Carbohydrate metabolism’ is another most abundant among the top five biological processes in feeding-specific, non-feeding-specific and upregulated transcripts in feeding adults (Table S7). Among the upregulated transcripts of this category in feeding aphids, three were putative Glycosyl hydrolase family 18 (GH18) members, two of which had signal peptide sequences. GH18 has earlier been identified as a potential effector in plant–fungus interactions [33]. Transcripts involved in carbohydrate metabolism and specific to feeding aphids were associated with enzymes, viz., Fructose-1, 6-bisphosphatase and Chitinase. Plant sap is rich in sugars, and hence, a higher expression of glycolytic enzymes such as Fructose-1 and 6-biphosphate for digestion of a carbohydrate-rich diet is expected.

Transcripts specific to non-feeding adults are involved in other metabolic processes, viz., glycogen phosphorylase activity and other carbohydrate metabolic process, indicating that, in non-feeding aphids, storage glycogen is broken down to glucose and mobilised for nutrition. Interestingly, chitinase activity, along with other energy metabolic processes, is accompanied by upregulation of the developmental gene *goosecoid*, a Homeobox protein gene. The activity of developmental genes may be explained as a probable consequence of starving ~300 aphids together for 3 h during sampling that may introduce starvation and crowding effects. Under such non-feeding conditions, the activation of morphogenesis genes to facilitate the development of winged forms and the upregulation of stress-responsive genes to overcome the resultant stress is expected to occur [60,61].

**Figure 6:**
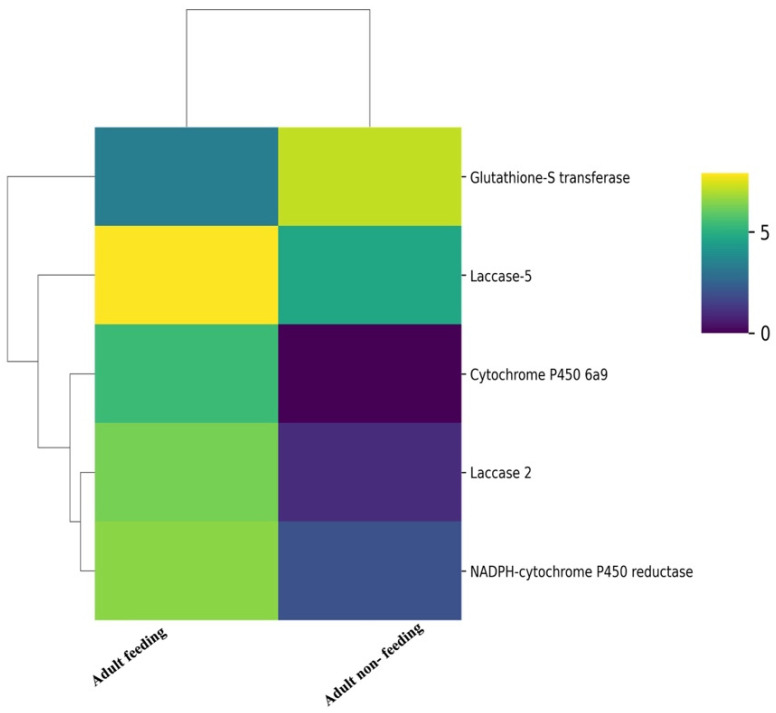
Heatmap with colour scales representing an unsupervised hierarchical clustering of differentially expressed transcripts between adult feeding and adult non-feeding involved in feeding-related functions: blue stars depict the category of detoxification, and orange stars depict the category of host recognition.

In order to identify other transcripts that are required for aphid feeding on host plants, we analysed the functional categories of the proteins involved in ‘detoxification’, which included detoxification-related proteins, viz, cytochrome P450 and laccase. We identified 14 cytochrome P450 transcripts and 4 NADPH-cytochrome P450 reductase transcripts in our transcriptome data. Only one NADPH-cytochrome P450 reductase transcript was upregulated in feeding adults with respect to non-feeding adults. Cytochrome P450 6a9 was specific to feeding aphids. Cytochrome P450 has been earlier shown to increase nicotine resistance in *M. persicae* [62]. The multi-copper binding enzyme Laccase, present in hemipteran salivary glands, oxidises plant phenolics [63]. *Laccase 1* has been shown to be upregulated in *A. glycines* fed on resistant plants [64]. We identified transcripts encoding other laccases, viz., laccase 2 (fragment), laccase 4, laccase 5 and laccase 25 in *L. erysimi*. Of the identified laccases, laccases 2 and 5 were significantly upregulated in feeding adults in comparison to non-feeding adults. Detoxification-related transcripts that were differentially regulated in feeding and non-feeding aphids are shown in a heatmap in Figure 6, and a complete list is provided in Table S8.

#### 3.3.3. Differentially Expressed Transcripts between Nymphs and Adults

**Figure 7:**
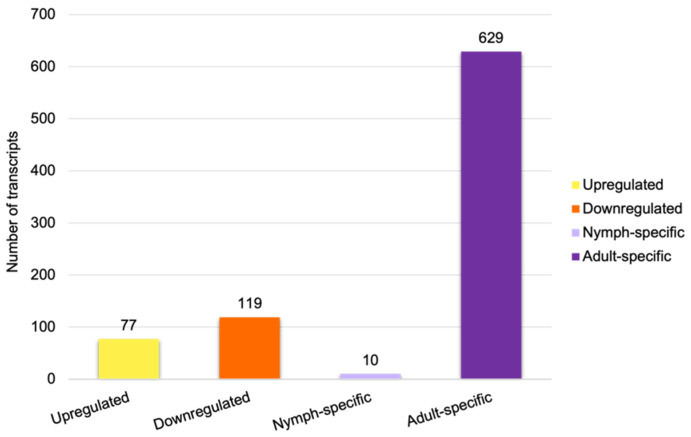
Number of differentially regulated transcripts between nymphs and feeding adults. Upregulated and downregulated transcripts represent the higher and lower expression levels in nymphs with respect to feeding adults.

A comparison of the nymph and adult transcriptomes identified 77 transcripts that showed significantly increased expression in nymphs (Figure 7; Table S9) and 119 transcripts with higher expression in adults (downregulated in NF vs. AF; Figure 7; Table S10). A total of 10 transcripts were specific to nymphs, and 629 transcripts were specific to adults (Figure 7 and Tables S11 and S12, respectively).

**Figure 8:**
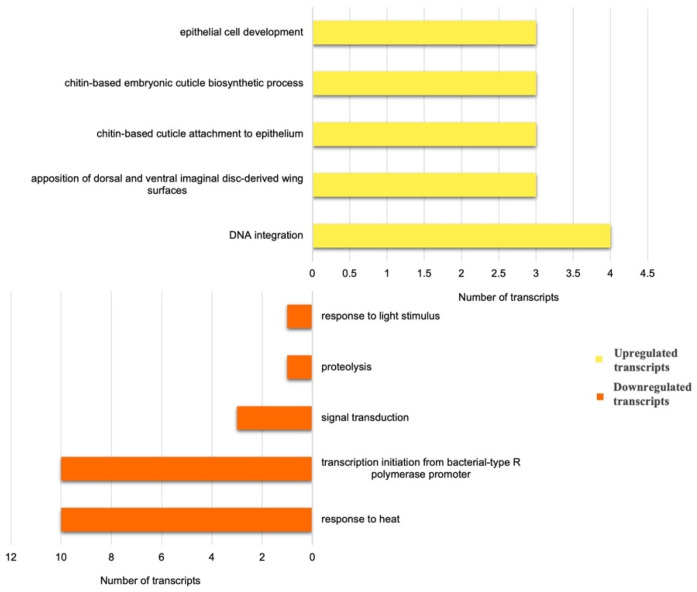
Classification of differentially expressed transcripts on the basis of Gene Ontology: Biological Process. The top 10 terms are represented for the comparison between NF vs. AF: Nymph Feeding vs. Adult Feeding. Upregulated and downregulated transcripts represent the higher and lower expression levels in nymphs with respect to feeding adults.

The major biological processes differing across the developmental stages are indicated by the five most abundant GO terms for biological processes for these transcripts (Figure 8). The upregulated nymph transcripts were predominantly involved in DNA integration, cytoskeletal organisation and developmental processes. Likewise, transcripts involved in GO biological processes—‘DNA integration’, ‘chitin-based cuticle biosynthetic process’, apposition of dorsal and ventral imaginal discs and microtubule cytoskeleton organisation—were upregulated in nymphs. Nymphs have actively dividing cells, since they undergo the process of ‘moulting’ (N1–N4 nymphal stages). This explains the upregulation of transcripts associated with the developmental processes and microtubule organisation, along with DNA integration and translation. DNA integration and chitin structuring were found to be most significantly influenced by the developmental stage of the aphids in this study, as seen in the case of the upregulation of these GO categories in nymphs with respect to the adult-feeding samples.

The 119 genes that were downregulated in nymphs in comparison to adults (i.e., upregulated in adults) were distributed across various biological processes, viz., the regulation of transcription, signal transduction and proteolysis, among others. The annotated downregulated transcripts included those coding for beta-glucanase and chemosensory protein for host recognition and others like cell wall-associated hydrolase, salivary protein and secreted proteins. This suggests that finding a suitable host and/or feeding site might be prominent roles for adult aphids rather than nymphs.

Interestingly, 36 upregulated transcripts in nymphs, including transcripts for development (dumpy isoform Z and O) and cytoskeleton organisation-related transcripts, were also upregulated in non-feeding adults when compared to the Adult Feeding samples. This might indicate the activation of developmental genes due to the onset of starvation, as discussed earlier. This overlap may also be due to the fact that moulting nymphs frequently stop feeding and, hence, may show effects similar to starvation [65].

### 3.4. Differential Expression of Putative Effectors

During feeding, aphids produce two kinds of saliva: gelling sheath saliva and liquid saliva. Gelling saliva protects the stylet and closes punctured cellular sites during its penetration, inhibiting the activation of plant defence responses [15]. Liquid saliva contains several proteins that function as ‘effectors’ and induce a plant defence response and also proteins that counter plant defences. To identify transcripts encoding putative effector proteins, we analysed the transcriptome using a bioinformatics pipeline that selects transcripts containing signal peptides and lacking a transmembrane domain or nuclear localisation signal. We identified 996 transcripts of putative effectors, of which 905 putative effectors were annotated with insect proteins. Forty-three transcripts did not identify with any of the reported insect proteins and, hence, could be concluded as ‘novel putative effectors’ identified in our study. Among the differentially expressed putative effectors between the adult-feeding and non-feeding states, four transcripts showed upregulated expression in the feeding aphids compared to non-feeding aphids. These included two transcripts encoding GH18 domain proteins and one transcript encoding a protein with identity to a mosquito protein with oxidoreductase activity. Both these classes of enzymes have been reported in aphid saliva and/or effectors in other aphids. Glycosyl hydrolase family 18 includes insect chitinases which orthologs have been reported in *A. pisum* [66]. Oxidoreductases (of the GMC family) have been identified in the saliva of *A. pisum*, *S. avenae* and *Metopolophium dirhodum* and have been implicated in the detoxification of plant defence molecules [67]. The total number of upregulated and specific putative effectors in adults and nymphs is given in Table 5, and the complete list of transcripts of putative effectors with annotation is given in Table S13.

Some of the putative effectors were also found to be developmentally regulated. A total of 16 putative effector transcripts were found to be differentially regulated between adults and nymphs. Among these, 8 putative effectors were upregulated in nymphs, 8 were upregulated in adults and 23 putative effector transcripts were specific to adults (Table 4). However, no nymph-specific putative effector transcripts were identified. These observations are in contrast to an earlier study by Elzinga et al. [68], wherein no significant differences in the expression of *M. persicae* effectors, viz, *MpC002*, *Mp1*, *Mp55*, *Mp56*, *Mp57* and *Mp58*, were found between adults and nymphs. Considering the differential regulation seen in other feeding-related transcripts, it is possible that aphid effectors might also be specific to a developmental stage. The higher frequency of effectors in adults as compared to nymphs also indicates their greater functional relevance in adults. It may also be due to the fact that nymphs emerge once the adults have settled on the host plant; therefore, nymphs do not engage much with host plants compared to adults. Differential expression of the putative effectors between nymphs and adults is being reported for the first time in our study.

### 3.5. Validation of Differentially Expressed Transcripts by qRT-PCR

**Figure 9:**
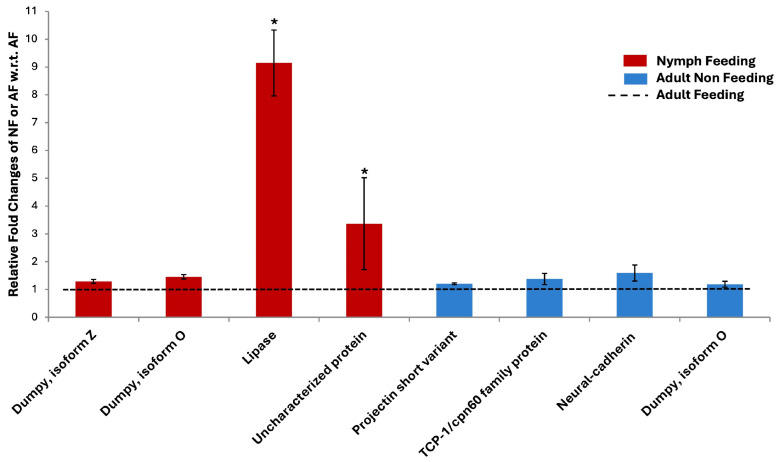
Graph representing the relative fold changes of selected differentially expressed transcripts using qRT-PCR. Relative fold changes were calculated using the *EF1α* gene for normalisation. To obtain relative expression data (∆∆Ct), the ∆Ct of each replicate of treatments AF (adult-feeding), NF (nymph-feeding) and ANF (adult non-feeding) was divided by the average ∆Ct of AF. The average of ∆∆Ct of each treatment was plotted in the graph by taking AF (adult feeding) as 1 (black-dotted line). Student’s *t*-test was conducted for statistical significance of the data. The asterisk mark (*) above data bars indicates statistically significant results at *p*_value_ ≤ 0.05.

For experimental validation using qRT-PCR of the digital expression data, i.e., expression profile obtained through transcriptome sequencing, we randomly selected eight transcripts with log2 fold changes ≥ 2 and ≤−2 across different samples. Their corresponding primer details are given in Table S1. Out of the eight selected transcripts, five transcripts (two transcripts of Dumpy isoform O, Dumpy isoform Z, neural cadherin and TCP-1/chaperonin) showed differential expression (P_adjusted_ value < 0.05). Pea aphid *Elongation Factor 1α* (*EF1α*) was used as an endogenous control for normalisation in qRT-PCR. For statistical significance in the qRT-PCR results, Student’s *t*-test was conducted. Of the eight total selected transcripts, two transcripts (Lipase and uncharacterised protein) showed significant results at *p*_value_ ≤ 0.05 (Figure 9). The qRT-PCR results for each selected target transcript were in consonance with the digital expression data. Dumpy isoform O, Lipase, uncharacterised protein and Dumpy isoform Z were upregulated in the nymph stage with respect to the adult stage. Another transcript of Dumpy isoform O, Projectin short variant, neural cadherin and TCP-1/chaperonin showed higher expression in the adult non-feeding with respect to adult feeding samples, thereby validating the starvation stress-induced upregulation of developmental genes, structural protein and chaperone.

Based on the results obtained in this study, we identified candidate genes associated with detoxification, metabolism and development (Table 6) that could be used for functional validation of their effects on aphid survival and/or fecundity using an RNAi approach. Promising genes can be deployed for the development of RNAi-based transgenic plants for aphid resistance. RNAi-based approaches have been shown to be effective against target pests and can contribute to environment-friendly agricultural practices by reducing the use of harmful agrochemicals, viz., insecticides and pesticides, and promoting organic farming [69,70,71].

The transcriptome data generated in this study augmented the existing genomic resources and identified, for the first time, variations in the global expression patterns of genes during feeding and non-feeding/starvation conditions in any aphid. Several of these differentially expressed transcripts are involved in host recognition, detoxification, defence response, nutrition and development, indicating significant differences and similarities in aphid physiology and behaviour under the studied conditions. These differentially regulated transcripts that are important for feeding and development can be utilised for designing effective aphid control strategies for *L. erysimi*. Many of the unannotated transcripts, which showed significant differential regulation during feeding or development, need to be functionally characterised to improve our understanding of their roles in aphid biology. Our transcriptome-based study of *L. erysimi,* a non-model aphid and a devastating pest of oilseed Brassicas in the Indian subcontinent under two different conditions, i.e., feeding and starvation, as well as two different developmental stages, has generated resources that would facilitate basic and applied research on aphid biology, evolution and control.

## Figures and Tables

**Table 1:** Sequencing data and quality analysis of the *L. erysimi* transcriptome.

Parameter	Adult Feeding (AF)	Adult Non-Feeding (ANF)	Nymph (NF)
Total Raw Reads	178,820,582	167,784,082	276,267,716
HQ Filtered Reads	165,696,944	155,298,042	215,410,036
Number of transcripts	51,491	51,715	49,868
Percent reads mapped to the transcriptome (%)	88.70	88.80	88.20

**Table 2:** Assembly statistics of high-quality reads.

Parameter	Primary Assembly Values	Final Assembly Values
Number of Final Transcripts	110,556	52,652
Final Transcriptome Length	89,232,845 (~89 Mbp)	56,027,788 (~56 Mbp)
Minimum Transcript Length	224	224
Maximum Transcript Length	26,824	26,824
Average Transcript Length	807.13	1064.12
N50	1475	1806
(G + C)%	34.75	34.52

**Table 3:** Results of the BUSCO analysis.

	Assembly Values
Complete BUSCOs	96.8%
Complete and single-copy BUSCOs	64.7%
Complete and duplicated BUSCOs	32.1%
Fragmented BUSCOs	1.0%
Missing BUSCOs	2.2%

**Table 4:** Top five KEGG pathways in order of transcript abundance.

KEGG ID	Pathway	No. of Transcripts
ko01100	Metabolic pathways	1123
ko03010	Ribosome	257
ko03013	RNA transport	190
ko03040	Spliceosome	138
ko04141	Protein processing in the endoplasmic reticulum	136

**Table 5:** Total number of upregulated putative effector transcripts in adults and nymphs.

	Number of ‘Upregulated’ Putative Effector Transcripts	Number of ‘Specific’ Putative Effector Transcripts
In feeding adults (with respect to non-feeding adults)	4	1
In feeding adults (with respect to nymphs)	8	23
In nymphs (with respect to feeding adults)	8	0

**Table 6:** List of potential candidate genes for functional validation and RNAi-mediated plant protection against aphids.

Transcript Id	Gene Ontology (Biological Process)	Expression Regulation
TR52991|c0_g1_i1	[GO:0005975] carbohydrate metabolic process	Not regulated with significant differences
TR59106|c0_g1_i1	[GO:0042744] hydrogen peroxide catabolic process [GO:0006979] response to oxidative stress	Not regulated with significant differences
TR35912|c0_g1_i1	[GO:0006979] response to oxidative stress	Adult specific (Nymph vs. Adult)
TR35912|c0_g2_i1	[GO:0006979] response to oxidative stress	Adult specific (Nymph vs. Adult)
TR35286|c0_g1_i1	[GO:0006979] response to oxidative stress	Upregulated (feeding adult vs. non-feeding adult)
TR35286|c0_g1_i2	[GO:0006979] response to oxidative stress	Not regulated with significant differences
TR35875|c0_g1_i1	[GO:0007475] apposition of dorsal and ventral imaginal disc-derived wing surfaces. [GO:0040005] chitin-based cuticle attachment to epithelium. [GO:0008362] chitin-based embryonic cuticle biosynthetic process. [GO:0002064] epithelial cell development. [GO:0046331] lateral inhibition [GO:0007424] open tracheal system development	Upregulated (Nymph vs. Adult)
TR35875|c0_g1_i5	[GO:0007475] apposition of dorsal and ventral imaginal disc-derived wing surfaces [GO:0040005] chitin-based cuticle attachment to epithelium [GO:0008362] chitin-based embryonic cuticle biosynthetic process [GO:0002064] epithelial cell development; [GO:0046331] lateral inhibition [GO:0007424] open tracheal system development	Upregulated (Nymph vs. Adult)
TR35875|c0_g1_i8	[GO:0007475] apposition of dorsal and ventral imaginal disc-derived wing surfaces [GO:0040005] chitin-based cuticle attachment to epithelium; [GO:0008362] chitin-based embryonic cuticle biosynthetic process [GO:0002064] epithelial cell development [GO:0046331] lateral inhibition [GO:0007424] open tracheal system development	Upregulated (Nymph vs. Adult)

## Data Availability

The sequencing data were submitted to the Sequence Read Archive of NCBI with accession number SRP093554.

## Supplementary Materials

The following supporting information can be downloaded at https://www.mdpi.com/article/10.3390/insects15090682/s1: Table S1: RT-PCR primers used in this study; Table S2: Total transcripts with annotation; Table S3: Total number of upregulated transcripts in feeding vs. non-feeding; Table S4: Total number of downregulated transcripts in feeding vs. non-feeding; Table S5: Total number of feeding specific transcripts; Table S6: Total number of non-feeding specific transcripts; Table S7: GO biological processes of differentially regulated selected transcripts; Table S8: List of DEGs involved in detoxification and host recognition; Table S9: Total number of upregulated transcripts in nymphs vs. adults; Table S10: Total number of downregulated transcripts in nymphs vs. adults; Table S11: Number of nymph-specific transcripts; Table S12: Number of adult-specific transcripts; Table S13: List of effectors.
